# Application of mesenchymal stem cell therapy for aging frailty: from mechanisms to therapeutics

**DOI:** 10.7150/thno.46436

**Published:** 2021-03-31

**Authors:** Yingqian Zhu, Jianli Ge, Ce Huang, Hailiang Liu, Hua Jiang

**Affiliations:** 1 Department of Geriatrics, Shanghai East Hospital, Tongji University School of Medicine, Shanghai, 200123, China.; 2 Department of General medicine, Shanghai East Hospital, Tongji University School of Medicine, Shanghai, 200123, China.; 3 Institute for Regenerative Medicine, Shanghai East Hospital, Tongji University School of Medicine, Shanghai, 200123, China.

**Keywords:** mesenchymal stem cells, aging frailty, aging, regenerative medicine, stem cell therapy

## Abstract

Aging frailty is a complex geriatric syndrome that becomes more prevalent with advancing age. It constitutes a major health problem due to frequent adverse outcomes. Frailty is characterized by disruption of physiological homeostasis and progressive decline of health status. Multiple factors contribute to development of frailty with advancing age, including genome instability, DNA damage, epigenetic alternations, stem cell exhaustion, among others. These interrelated factors comprehensively result in loss of tissue homeostasis and diminished reserve capacity in frailty. Therefore, the aged organism gradually represents symptoms of frailty with decline in physiological functions of organs. Notably, the brain, cardiovascular system, skeletal muscle, and endocrine system are intrinsically interrelated to frailty. The patients with frailty may display the diminished reserves capacity of organ systems. Due to the complex pathophysiology, no specific treatments have been approved for prevention of this syndrome. At such, effective strategies for intervening in pathogenic process to improve health status of frail patients are highly needed. Recent progress in cell-based therapy has greatly contributed to the amelioration of degenerative diseases related to age. Mesenchymal stem cells (MSCs) can exert regenerative effects and possess anti-inflammatory properties. Transplantation of MSCs represents as a promising therapeutic strategy to address the pathophysiologic problems of frail syndrome. Currently, MSC therapy have undergone the phase I and II trials in human subjects that have endorsed the safety and efficacy of MSCs for aging frailty. However, despite these positive results, caution is still needed with regard to potential to form tumors, and further large-scale studies are warranted to confirm the therapeutic efficacy of MSC therapy.

## Introduction

The global population is aging rapidly due to an increase in life expectancy 1, so too has the increasing prevalence of aging frailty 2. Frailty is an age-associated geriatric syndrome, defined as a state of increased physiological vulnerability to stressors due to multiple system dysregulation and reduced functional reserves 3. Aging frailty is associated with functional limitations in daily living, which conferred the greater risk of poor health outcomes in the older population, such as mortality, disability, hospitalization and falls 4-6, alongside the increased healthcare costs which presents a major public health problem worldwide 7, 8. Despite decades of research that have led to a growing understanding of biological alterations of frailty, the approved medical therapy that can effectively attenuate or reverse aging frailty is still not available 9. To date, clinicians have attempted several interventions to improve and modify frailty status, including physical exercises (e.g., strengthening exercises), nutrition (e.g., protein and Vitamin D), and multidisciplinary interventions 9, 10, but the efficacy of these interventions for protecting the frail patients against adverse outcomes is still controversial 11, 12. Since frailty is one of the biggest threats to successful aging, a specific intervention that is expected to be effective to improve frailty status is highly needed. Currently, cell-based therapy is emerging as an innovative approach for several degenerative diseases. Mesenchymal stem cells (MSCs) represent as the ideal seeding cells for tissue engineering and regenerative medicine 13, 14. To date, MSCs has become a promising candidate for intervening aging frailty. In this review, we mainly focused on the pathobiological process of aging frailty and summarized the roles and mechanisms of MSCs as the novel biologic agents used in the treatment of aging frailty. We also discussed the current status of MSCs utilized in clinical research as well as the challenge for successful clinical applications of MSC therapy.

## Overview of Aging Frailty and its Pathophysiology

Aging frailty is a complex geriatric syndrome with multifactorial pathogenesis and declines in physiological reserves. Frail syndrome can lead to the reduced homeostatic capability to withstand stressors and increased vulnerabilities to environments, which account for the high risk of adverse events 2, 15, 16. The overall prevalence of aging frailty in community worldwide is estimated to be between 5% and 20% 17-19. The prevalence of frailty increases with age and women are more likely to be frail than men 18. Aging frailty can be identified by two main models: physical frail phenotype and cumulative deficit index 2, 15. According to the phenotypic model, frailty can be identified by the presence of at least three components: unintentional weight loss; self-reported exhaustion; weakness; slow walking speed and low physical performance 2. It is characterized by diminished strength, endurance and reduced physiologic function, which increase an individual's vulnerability for developing increased dependency or death 20. On the other hand, the deficit model describes frailty in terms of the accumulation of individual impairments that include comorbid diseases, symptoms, signs and disabilities, collectively referred to as deficits 15. While these two instruments are different for evaluating frailty, both have received empirical validation.

With the process of aging, frailty may be caused by multiple causes and contributors, including genetic and environmental factors 21-24. To be more specific, genome instability 25, DNA damage 26, epigenetic alternations 27, loss of proteostasis 28, oxidative stress 29, chronic inflammation 30, mitochondrial dysregulation 31, and stem cell exhaustion 24, 32 are involved in the progression of aging frailty. These hallmarks are interconnected and ultimately lead to cellular senescence. The senescent cells increase in multiple tissues with aging 33, and secrete a host of inflammatory cytokines, chemokines, growth factors and matrix remodeling proteases, collectively known as the senescence-associated secretory phenotypes (SASP), which lead to the chronic inflammation and age-related tissue deterioration 34, 35. Moreover, senescence reduces the regenerative potential of stem cells pools and leads to endogenous stem cells exhaustion. The resident stem cells, including MSCs, HSCs (hematopoietic stem cells), neural stem cells (NSCs) and satellite cells undergo senescence during aging process, showing age-related decline in repopulation capacity and differentiation potential with reduced lifespan 36-39. The reduced abilities of stem cells fail to maintain their proliferation capacity and differentiation potential 40. Accordingly, the capacity to regenerate damaged tissues decline of regeneration upon damage decline, which results in the imbalance of tissue homeostasis after injury or stress 34, 41. The sum of these integrative hallmarks produces the clinical phenotypes of the elderly with aging frailty, as seen in physiological loss of reserve and reduced organ function 42. The disfunctions of brain, heart, muscle, and endocrine system are linked to aging and impaired homeostasis, which are believed to be involved in the development of frailty 16. The multiple types of aging-related damages may constitute the major culprits of phenotypes of frailty, as the integrative consequence of stem cell exhaustion, diminished homeostasis, and organ repair 43. In this regard, regenerative medicine and cellular therapy has been long proposed and examined clinically. As a promising candidate for tissue regeneration, MSCs have gathered great attention in the field of regenerative medicine. Transplantation of MSCs may serve as an innovative therapeutic approach for preventing and even reversing development of aging frailty 44, 45.

## Basic Characteristics of MSCs

MSCs are the non-hematopoietic stem cells which exhibit spindle-shaped structure and plastic-adherent properties 46. Originally isolated from bone marrow in 1968 47, MSCs were successively found to exist in various tissues and can be easily harvested from multiple tissues, including adipose tissue, marrow spaces of long bone, skeletal muscle, synovial fluids, umbilical cord blood, placenta, and dental pulp 48-51. As the multipotent progenitors, MSCs have displayed the ability to give rise to several different phenotypes, including osteocytes, chondrocytes, adipocytes, fibroblast, and many others 52, 53. However, MSCs exhibit heterogeneous features among their subpopulations regarding to their proliferation rate and secreted cytokines 54, 55. In addition, the discrepancy of isolation and cultivation procedures between different laboratories also drives the development of standardized criteria for identifying unique populations of MSCs. In 2006, the International Society for Cellular Therapy (ISCT) has proposed the minimum criteria to define human MSCs 46. According to ISCT, MSCs must be plastic-adherent and positive for specific surface makers, namely, CD73, CD90 and CD105 but be negative for CD14, CD19, CD34, CD45 and HLA-DR. More importantly, MSCs must be capable of differentiating into multilineage cell types *in vitro*. MSCs can migrate automatically toward injury areas and spontaneously differentiate into desired tissues to perform regenerative functions, which are described as tropism 48, 56. The therapeutic effects of MSCs, including their anti-inflammatory and immunomodulatory abilities, are exerted via secretion of several cytokines and soluble factors and signaling pathway activation. MSCs had the low expression of MHC/HLA class I but do not express MHC/HLA class II, which can protect them from host immune detection. The biological property of immune evasion prolongs their persistence in the host and enhances their therapeutic effects 57. To date, MSCs have been considered as one of the most promising stem cell types for cell therapy. MSCs are associated with unique capability of self-renewal and extensive potential of differentiation, which have generated great interest in the fields of regenerative medicine 58. Multiple lines evidence have documented that the transplantation of MSCs can be utilized as a suitable therapeutic approach in the treatments of some intractable diseases, including traumatic brain injury 59 and spinal cord injury 60, cardiovascular diseases 61, stroke 62 and liver diseases 63. The specific characteristics, along with the therapeutic benefits of MSCs support the potential use of MSCs in future therapies for aging frailty.

## MSC Therapy for the Attenuation of Aging Frailty

### Aging Brain

Frailty is a state of increased vulnerability to stressor events due to multimorbidity and multiple impairments in different systems. Aging brain or frail brain would lead to central nervous system impairments with cognitive decline, which play a crucial role in the development of physical frailty 64, 65. More importantly, the deterioration of brain is associated with gait impairments, which is considered as an important contributor to frailty 66.

### Neuroprotective Effects of MSCs

**Figure 2:**
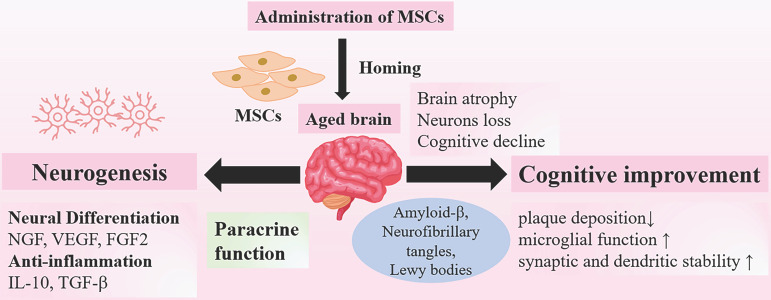
** Neuroprotective Effects of MSCs on Aging Brain.** Administration of MSCs have shown therapeutic potential for the treatment of age- related brain dysfunction. The neuroprotective effects of MSCs include promoting neurogenesis, neural differentiation and anti-inflammation, and these effects are mainly associated with paracrine functions. In addition, MSCs could improve cognitive functions through reducing plaque deposition and enhancing synaptic stability. Abbr: NGF: nerve growth factor; VEGF: vascular endothelial growth factor; FGF2: fibroblast growth factor 2; IL-2: interleukin-2; TGF-β: transforming growth factor-β.

In aging process, almost all the brains undergo characteristic changes, including brain atrophy, loss of neurons and synapse connections. These age-related changes are responsible for the decline in neuronal activity and synaptic dysfunction that linked to neurodegeneration 67. The effects of transplanted MSCs have been documented *in vivo* and vitro experiments in several studies, which have shown that MSCs could promote neurogenesis and improve neurological state 68, 69. Intravenous infused MSCs can cross the blood-brain barrier (BBB), which is an essential prerequisite for proper efficacy 70-72. Then intravenous injected MSCs can migrate to the injured regions and differentiate into neuron-like-cells via secreting various neurotrophic factors, such as nerve growth factor (NGF), vascular endothelial growth factor (VEGF) and fibroblast growth factor 2 (FGF2). These secretomes are released from non-genetically modified MSCs, playing a significant role in inducing neuronal differentiation and increasing survival rates after injury 73, 74. Likewise, administration of MSCs via intracerebral and intrathecal routes also showed positive results of neuronal regeneration promoted by MSCs in animal models 75, 76. Moreover, microglia and astrocytes in aging brain become senescent and express the senescence-associated secretory phenotype; several inflammatory cytokines are secreted to maintain state of low-grade inflammation that play a significant role in natural aging and neurodegeneration 77. MSCs possess anti-inflammatory properties adding to their neuroprotective effects. A great number of studies have showed that transplanted MSCs could reduce the levels of pro-inflammatory cytokines 78, or promote macrophages to polarize into the anti-inflammatory M2 phenotype 79. The anti-inflammatory effects are conducted through secreting multiple cytokines, including IL-10 and transforming growth factor-β (TGF-β) 80. At such, the anti-inflammatory microenvironments induced by transplanted MSCs help promote neurogenesis and prevent neural degeneration 78, 81. Ameliorating cognitive decline may be a promising approach to prevent brain frailty. There are several altered proteins in the aged brains. The presence of amyloid-β, neurofibrillary tangles, Lewy bodies, the causative factors of neurodegenerative diseases, such as AD, may contribute to deterioration of brain 66, 82, 83. Inspiringly, MSCs administration has been documented to reduce plaque deposition, restore microglial function and increase synaptic and dendritic stability in animal models of AD 68, 69. To date, substantive preclinical studies are underway to provide positive results, and MSC-based therapy carries promise to reverse the deterioration of brain, which has become a potential therapeutic approach for the amelioration of aging frailty (Figure 2).

### Cardiovascular Risk

The cardiovascular diseases and aging frailty often coexist. Growing evidence has showed that cardiovascular diseases including myocardial infarction, atrial fibrillation and chronic heart failure are associated with the increased high incidence of aging frailty 84-86. Cardiovascular diseases could give rise to physical disability and frailty through impaired muscle function 85, 87. The interplay between cardiovascular diseases and frailty may provide a novel therapeutic strategy in the interventions of frailty.

### Cardioprotective Effects of MSCs

In the aging process, aging is associated with the gradual loss of biological functions, resulting in the increased cardiac vulnerability to cardiovascular dysfunction. The cardiac senescence is reflected by decreased cardiac performance and progressive cardiac structural remodeling. The various phenotypic changes in functions and structures of heart, including cardiomyocyte hypertrophy and apoptosis 88, interstitial fibrosis 89, comprehensively account for the decreased cardiac function, which may eventually lead to the progression of cardiovascular diseases in the aging populations. Several preclinical studies have demonstrated that MSCs could exert cardio-protective effects and promote cardiac functions through different mechanisms. MSCs could migrate to the injured zone and differentiate into endothelial cells and cardiomyocyte-like cells to promote neovascularization and cardiac functions, which can effectively offer repair in the sites of damaged myocardium. It has been found that MSCs exert many therapeutic functions through paracrine effects 90, 91. MSCs can produce multiple cytokines and angiogenic factors released directly in soluble form or in extracellular vesicles and exosomes, playing a role in improving cardiac functions after damage 92. The left ventricular ejection fraction (LVEF) is a significant parameter for evaluation of cardiac function, which would become deteriorated subsequently after ischemic events. It has been shown that LVEF can be successfully preserved in the MSCs treated group as compared to the control group in the animal model with ischemic myocardium 93. The positive results have been further confirmed in clinical trials that transplantation of MSCs could significantly attenuate adverse ventricular remodeling and improve LVEF in patients with heart failure 94, 95. Furthermore, many other studies have demonstrated MSC therapy can be capable of reducing the infract size and promoting cardiac hemodynamics in mice with ischemic myocardium 96. Current evidence shows that MSCs could persist for 4 weeks after transplantation, predominantly in the border zone of infarcted myocardium, whereas few MSCs were detected in the normal cardiac tissues 97.

**Figure 3:**
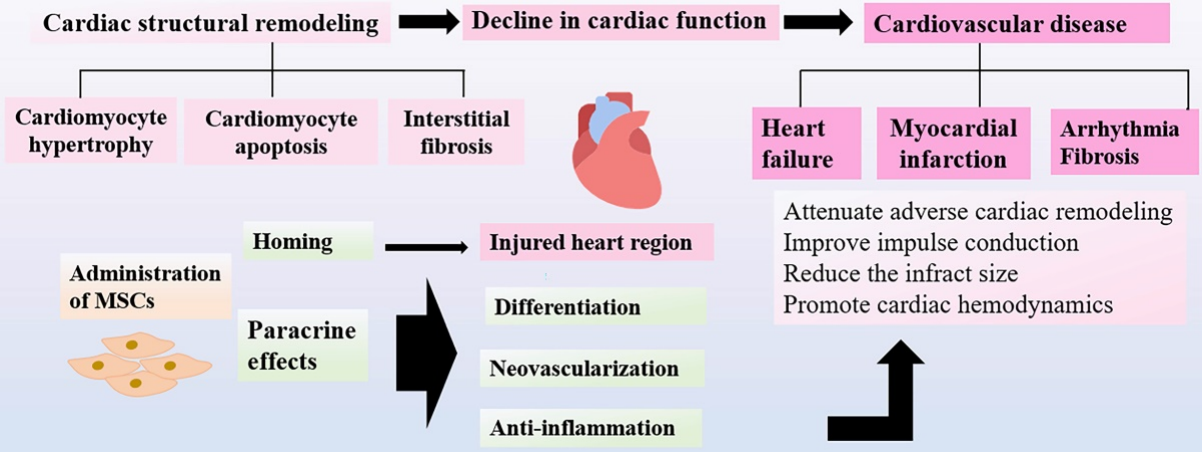
** Cardioprotective Effects of MSCs.** With advancing age, heart often develop decreased cardiac performance. The progressive cardiac structural remodeling results in low cardiac function and cardiovascular diseases that may contribute to aging frailty. After administration, MSCs home to the injured regions, where MSCs differentiate into endothelial cells and cardiomyocyte-like cells to promote neovascularization and cardiac functions. MSCs can suppress the excessive inflammatory responses and subsequent fibrosis via paracrine functions. MSC therapy show positive results by improving the prognosis of cardiovascular diseases.

It has been well recognized that fibroblast could replace cardiomyocytes after injury, which cause myocardial remodeling and fibrotic scarring. The anti-fibrotic molecule, TNF-α-induced protein 6 (TNAIP6) is secreted by MSCs to decrease the damage to the heart and fibrosis. MSCs suppress the excessive inflammatory responses caused by cardiomyocyte cells injury and subsequent fibrosis 98. In addition, MSCs attenuate arrhythmia by improving impulse conduction in the model of myocardial infarction 99. Taken together, this novel approach of MSCs transplantation can ameliorate cardiovascular symptoms via several mechanisms, including angiogenesis, repair of the injured tissue, and reduction of infarct size as well as regulation of cardiac structural remodeling, which has a great potential to be applied in the regenerative medicine to improve the treatment of aging frailty 100 (Figure 3).

### Sarcopenia

Sarcopenia is an age-related disease with the progressive loss of muscle mass and strength 101, 102. The declines in skeletal mass and function pose significant risks for adverse outcomes including mortality, disability and falls among older adults 103-105. The identification of sarcopenia is based on the co-occurrence of low muscle mass as well as slow gait speed or weak handgrip strength as measures of low muscle function 106. Sarcopenia has been considered as an important component of frailty syndrome and the pathway through which the frail condition can be intervened or reversed 107.

### Protective Effects of MSCs on Muscles

**Figure 4:**
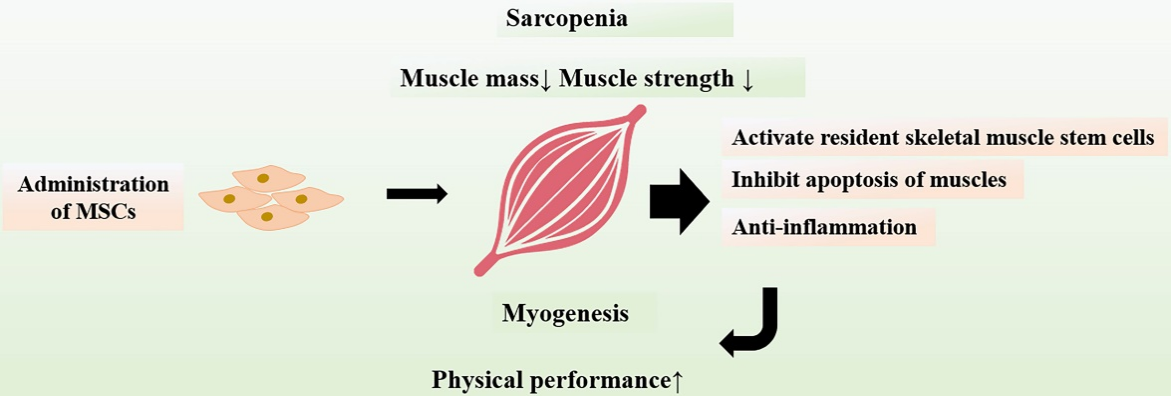
** Protective Effects of MSCs on Muscles.** Sarcopenia is a major contributor to frailty in the elderly. Transplanted MSCs can exert protective effects on muscles, including inhibition of muscles apoptosis and regulation of chronic inflammatory as well as activation of resident skeletal muscle stem cells. Administration of MSCs can promote myogenesis and improve physical performance.

The interventions that can alleviate sarcopenia may be an important approach to improve or reverse frailty status. It has been showed that MSCs could attenuate sarcopenia via increasing skeletal muscle weight and myofiber cross-sectional area in animal models of sarcopenia 108. The physical performance including muscle strength as well as endurance were significantly enhanced. MSCs also inhibit apoptosis of muscles and suppress expressions of chronic inflammatory cytokines, which may explain the improvement of skeletal muscle strength and function after transplantation of MSCs. In addition, MSCs have capability to activate resident skeletal muscle stem cells, which lead to myogenesis and differentiation of muscle tissues 109. The positive results provide novel insights into sarcopenia intervention, suggesting a potential role for MSC therapy in aging frailty (Figure 4).

### Altered Hormones

Advance in age leads to the disruption of endocrine system and imbalance of metabolic homeostasis, which may result in the breakdown of adaptation process in response to stresses 110. The alternations in hormonal networks and abnormal hormonal excesses or deficits during aging can be translated in clinical scenarios that promote the pathogenesis of frailty and diseases 111. As age-related disruption of the endocrine system is considered as a fundamental event in the pathogenesis of frailty, the efficacious strategies that can promote metabolism are needed.

### Therapeutic Effects of MSCs on Hormones

Accumulating evidence shows that adverse ageing profiles and frailty are related to the alternations in hormonal networks 110-112. Age-related frailty is a common problem in older adults, as a result of the imbalance between the anabolic and catabolic hormones. The circulating anabolic hormones, including insulinlike growth factor (IGFs), growth hormone, and sex hormones, are important in maintaining healthy body compositions and organ functions. However, there is an overall decline in the amounts of hormones with age. For instance, the decreased levels of testosterone could lead to hypogonadism and reduced muscle mass. Researchers have documented that MSCs transplantation could recover the levels of testosterone back to normal through paracrine functions 113. Notably, growth hormone and IGF1 also decrease with aging, the insufficient hormones result in body composition parameters with elevated fat mass and reduced lean mass 110, 114. MSCs exerting beneficial paracrine effects are well recognized. It has been found that MSCs are capable of secreting multiple growth factors and cytokines, promoting regeneration of Leydig cells and many surrounding cells 113, 115. In addition, MSCs can develop and differentiate into Leydig cells in the adult testis 116.

**Figure 5:**
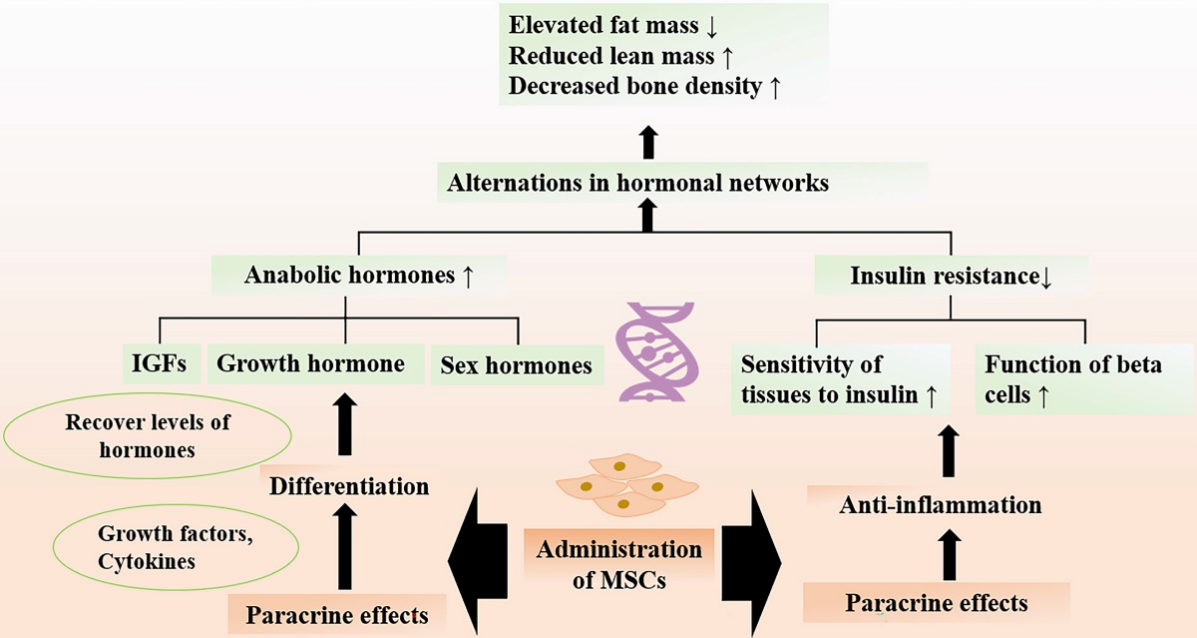
** Therapeutic Effects of MSCs on Abnormal Hormones.** Age-related alternation in hormonal networks include the decline in levels of circulating anabolic hormones and insulin resistance, which are associated with development of frailty. Administration of MSCs can increase the levels of anabolic hormones through paracrine functions and improve insulin sensitivity by regulating immune response. MSC therapy attenuate the age-related structural and functional changes of muscle and bone, thereby promoting the quality of life among the older adults.

In addition to the deficiency of hormones, decreased sensitivity of tissues to actions of hormone take place in the elderly. Notably, insulin resistance develops with age, which is a state of poor sensitivity of peripheral tissues to insulin 117. Insulin resistance may lead to metabolic disorders and accelerate decline in muscle strength and function that give rise to frailty 118, 119. The roles of aging endocrine system in the development of frailty and as a target for interventions of frailty are investigated. The chronic inflammation is an important determiner of insulin resistance 120, so the protective role of MSCs in improving insulin sensitivity via suppressing the inflammatory activity has been focused. Preclinical study showed that MSCs after transplantation could significantly promote the response of target organs to insulin 121. The therapeutic effect of MSCs may be attributed to regulation of immune process and systemic inflammation 122. Numerous data have reported that MSC-based therapy can attenuate insulin resistance and improve beta cell function via inhibiting the production of inflammatory cytokines (e.g., IL-1β, Il-18, TNF-α) 123. MSCs play a pivotal role in reducing the number of CD3+ and CD4+ T lymphocytes, which initiate the inflammatory process in the organism 122. Given the therapeutic potential of MSCs on delineating the age-related alterations of hormones, MSC-based therapy may be a very promising candidate for promoting quality of life in the elderly population (Figure 5).

## Clinical Transplantation of MSCs in Patients with Aging Frailty

While current evidence sheds a promising light for the stem cell-based therapy, data related to frailty is still limited in clinical settings 124, 125. Aging FRailTy via IntravenoUS Delivery (CRATUS) went through the phase I and II stages. The phase I trial was a nonrandomized, dose-escalation study, which has reported the beneficial effects after transplantation of BM-derived MSCs in patients with aging frailty 124. In that study, a total of 15 eligible patients were enrolled to receive the intravenous infusion of MSCs with the dose: 20-million, 100-million, 200-million, respectively (5 patients in each group). Inspiringly, all patients in the treatment groups had increased 6-minute walk distance at 3 months and 6 months. The levels of inflammatory cytokine, TNF-α decreased at 6 months. Among the three groups, 100-millon cell-dose group showed the best performance in the improvement of 6-minute walk distance, cognitive status and physical function. With regard to the safety of MSCs administration, no treatment-emergent serious adverse events occurred within 1-month post infusion. All patients could tolerate the doses of MSCs infused well. One death was reported at 258 days after infusion in the 200-million group which was determined to be irrelevant to MSCs transplantation. This study above-mentioned was succeeded by the randomized, double-blinded, and placebo-controlled, stage II of CRATUS study 125. In the consecutive study, a total of 30 patients with aging frailty were randomized into 100-million, 200-million, and placebo groups. The results showed that immunologic improvement was seen in both the treatment groups. Notably, patients in the 100-million group performed better than that in the 200 million with improved 6-minute walk distance, short physical performance, forced expiratory volume in 1 second and decreased serum TNF-α levels from baseline to 6 months. More importantly, this study documented that intravenous administration of MSCs was safe, which did not incur any treatment-related serious adverse events for 12 months post infusion. Intriguingly, the consecutive two trials confirm that 100-million cells represent the superior dose level compared to 200-millon cells, yet the mechanism underlying the inverse dose relationship cannot be sufficiently explained 125. A plausible explanation may be associated with deleterious effects of higher doses on cell retention, survival, or performance. Despite the positive findings, these two trials are preliminary and require larger RCTs to yield more convincing conclusions.

In recent years, failure of MSCs to improve clinical outcome have been frequently encountered 126, 127, partially due to variability in culture methodologies 128, and poor survival of MSCs after transplantation 129. The effect of MSCs largely depends on their capabilities to migrate, adhere, engraft to the injured site. Notably, the freshly isolated cells cultured in presence of specific cytokines or hypoxic conditions have higher engraftment efficiency 130. Furthermore, aggregate culture conditions used for MSC production may improve secretory capacity 128. The use of different MSC derivatives, such as extracellular vesicles and exosomes, may be more effective and preferable than the use of MSCs. There is still a long way to go before considering MSCs as an ideal clinical tool for aging frailty.

## Challenges for Clinical Application of MSC Therapy

### Efficacy

MSCs have the distinct advantages of rapid expansion, multi-lineage differentiation and potent ability of secreting tropic and immunomodulatory cytokines. For years, transplantation of MSCs has evolved as the promising therapeutic strategy for regenerative medicine and tissue engineering 131. However, there are major limits to MSCs utilization. The allogeneic MSCs derived from different donors display different biological properties. Aged MSCs tend to exhibit the cellular senescence associated phenotypes, including the enhanced senescence-associated β-galactosidase activity, decreased stemness of stem cells, increased p16 expression, and apoptosis of cells as well as telomere attrition 132, 133. With telomeres shortening, aged MSCs gradually cease to proliferate after a certain number of cell divisions. The proliferation and differentiation potential of MSCs progressively decline with age of donor and passage number of MSCs cultured *in vitro* 134, 135. Cellular senescence impairs the self-renewal and differentiation potential of MSCs, which limit their therapeutic effects 136. The replicative senescence of MSCs significantly limits their expansion to the large quantity necessary for clinical applications that need hundreds of millions of MSCs for per treatment 137. Moreover, there are limits for autologous MSC applications. It is difficult to obtain sufficient amount of healthy MSCs from patients with some systemic diseases. Additionally, the process of autologous extraction is time-consuming, which is difficult to be utilized for the acute treatment of life-threatening diseases 131. Other concerns regarding the efficacy of MSCs are their persistence after transplantation. These issues need to be addressed prior to widespread clinical application to enhance the efficacy of MSC therapy.

### Safety Concerns

MSCs are emerging as the promising sources of cell-based therapy due to their pluripotency and ease of expansion. However, ethical issues regarding to security remain inadequately addressed. It has been noted that long-term MSC expansion *in vitro* can lead to chromosomal abnormalities 138, 139, which may induce tumors *in vivo* 140. In the tumor microenvironment, MSCs possess immunosuppressive effects, which promote the progression of tumors 141, 142. MSCs show the potential to differentiate into multiple tissues, such as bone and cartilage, so the unwanted differentiation of transplanted MSCs may promote tumor growth 143. Furthermore, it is well accepted that angiogenesis exerts an important role in invasion and metastasis of tumors. MSCs can differentiate into vascular endothelial cells, secreting several growth factors including VEGF and PDGF (platelet-derived growth factor), which promote tumor angiogenesis and invasive behavior 144. MSCs also involve in the tumor invasion and metastasis known as epithelial to mesenchymal transition (EMT), a process driving tumor cells to lose polarity and acquire invasive phenotype 145, 146. In this regard, the tumorigenic potential of MSCs may become a major safety concern for the use of MSCs in clinical practice. MSC-based therapy may be a double-edged sword; the application of MSCs in clinical setting should be evaluated cautiously due to security concerns. Of note, as paracrine effect of MSCs plays a pivotal role, the bioactive secretions of MSCs have good efficacy and safety. For instance, extracellular vesicles, exosomes, and cytokines can avoid the risk of genetic instability and potential malignant transformation may be developed as a safe and effective agent in the regenerative medicine.

## Conclusions and Future Perspectives

Frailty syndrome is a nonspecific state of increased vulnerability to stressors and is much more common in the old populations. Frailty is strongly associated with adverse outcomes, which may place a heavy burden on society in the coming years. As there is no specific approved treatment for frail patients, deeper understanding of the biological mechanisms of aging frailty to explore effective interventions is of great significance. Notably, multiple pathologic changes develop with age, aside from DNA damage and chronic inflammation that may contribute to aging frailty, endogenous stem cell exhaustion may be involved in the process of aging frailty. The frail patients may display the disruption of physiological homeostasis with decline in functions of several organs.

MSCs are emerging as the ideal sources of cells to solve the multi-organ problems. MSCs have potent self-renewal and differentiation capability. They are easy to be harvested from many tissues and can engraft to injured sites. In addition, the immune privileged state and anti-inflammatory property make MSC-based therapy as a promising tool in systemic applications. Current evidence has showed that MSCs could ameliorate status of frailty by promoting the functions of multiple important organs, including brain, muscles, heart, and endocrine system. To date, allo-hMSCs had undergone the phase I/II trials in which the safety and efficacy of MSC-based therapy for aging frailty were initially demonstrated. MSCs could attenuate symptoms of frail patients and no treatment-related serious adverse event was reported.

Transplantation of MSCs has generated great interests in regenerative medicine. However, the disputes arise regarding lack of efficacy as well as tumorigenic potential of MSCs on basis of current evidence. Although many findings shed a new light on MSC-based therapy for aging frailty, the scales and numbers of current clinical trials remain small, much further studies are warranted to elucidate if such therapeutic strategy could be safe and effective on regenerative medicine. The underlying mechanism of MSCs transplantation for the intervention of aging frailty should also be investigated.

## Figures and Tables

**Figure 1:**
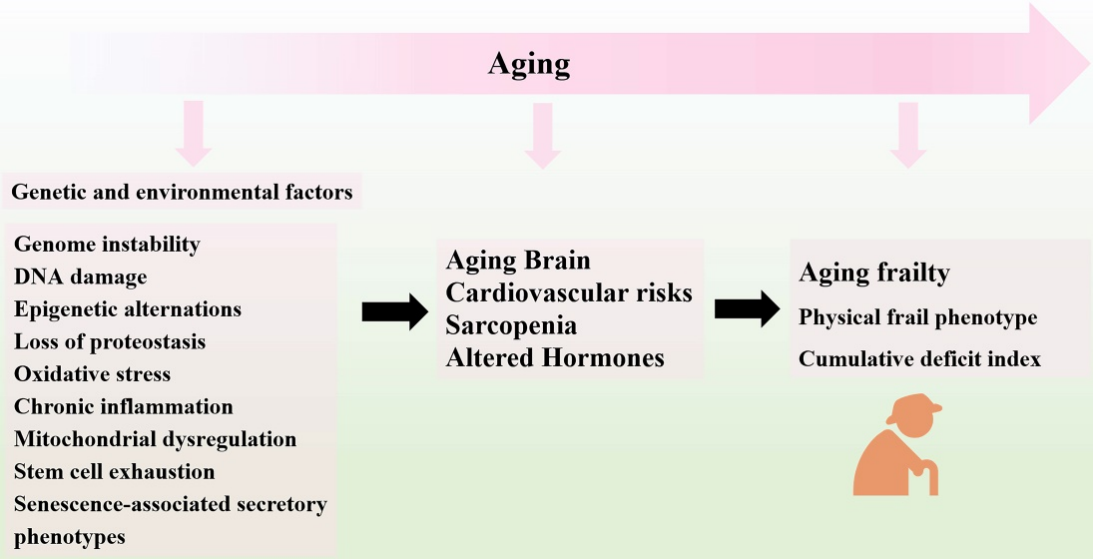
** Overview of Aging Frailty and its Pathophysiology.** In the process of aging, several genetic and environmental factors gradually result in the loss of tissue homeostasis and organ dysfunction, ultimately leading to the progression of frailty in the aged organism.
